# GBDT_KgluSite: An improved computational prediction model for lysine glutarylation sites based on feature fusion and GBDT classifier

**DOI:** 10.1186/s12864-023-09834-z

**Published:** 2023-12-11

**Authors:** Xin Liu, Bao Zhu, Xia-Wei Dai, Zhi-Ao Xu, Rui Li, Yuting Qian, Ya-Ping Lu, Wenqing Zhang, Yong Liu, Junnian Zheng

**Affiliations:** 1 School of Medical Informatics and Engineering, Xuzhou Medical University, Xuzhou, Jiangsu 221004 China; 2 Cancer Institute, Xuzhou Medical University, Xuzhou, Jiangsu 221004 China; 3 Jiangsu Center for the Collaboration and Innovation of Cancer Biotherapy, Xuzhou Medical University, Xuzhou, Jiangsu 221004 China; 4 School of Life Sciences, Xuzhou Medical University, Xuzhou, Jiangsu 221004 China; 5 School of Humanities and Arts, China University of Mining and Technology, Xuzhou, Jiangsu 221116 China; 6 Center of Clinical Oncology, The Affiliated Hospital of Xuzhou Medical University, Xuzhou, Jiangsu 221002 China

**Keywords:** Lysine glutarylation, Post-translational modification, GBDT, Elastic Net, NearMiss-3

## Abstract

**Background:**

Lysine glutarylation (Kglu) is one of the most important Post-translational modifications (PTMs), which plays significant roles in various cellular functions, including metabolism, mitochondrial processes, and translation. Therefore, accurate identification of the Kglu site is important for elucidating protein molecular function. Due to the time-consuming and expensive limitations of traditional biological experiments, computational-based Kglu site prediction research is gaining more and more attention.

**Results:**

In this paper, we proposed GBDT_KgluSite, a novel Kglu site prediction model based on GBDT and appropriate feature combinations, which achieved satisfactory performance. Specifically, seven features including sequence-based features, physicochemical property-based features, structural-based features, and evolutionary-derived features were used to characterize proteins. NearMiss-3 and Elastic Net were applied to address data imbalance and feature redundancy issues, respectively. The experimental results show that GBDT_KgluSite has good robustness and generalization ability, with accuracy and AUC values of 93.73%, and 98.14% on five-fold cross-validation as well as 90.11%, and 96.75% on the independent test dataset, respectively.

**Conclusion:**

GBDT_KgluSite is an effective computational method for identifying Kglu sites in protein sequences. It has good stability and generalization ability and could be useful for the identification of new Kglu sites in the future. The relevant code and dataset are available at https://github.com/flyinsky6/GBDT_KgluSite.

**Supplementary Information:**

The online version contains supplementary material available at 10.1186/s12864-023-09834-z.

## Background

Protein post-translational modification (PTM) is crucial in controlling the biological function of proteins and the molecular foundation for protein dynamic reactions and interactions, at the same time, it is also an important target for the regulation of cellular signaling networks. Lysine glutarylation (Kglu) was first reported by Tan et al. in 2014 [1], which refers to the process of covalently binding glutaryl groups (such as glutaryl-CoA and other donors) to lysine residues of substrate proteins under the catalysis of enzymes. So far, lysine acetyltransferase p300 and KAT2A (lysine acetyltransferase 2A) have also been reported to perform glutaryl-transferase function, while SIRT5 and SIRT7 are responsible for catalyzing the deglutarylation process. The Kglu modification changes the positive charge in lysine to a negative charge, which affects protein conformation and protein–protein interactions. Kglu has important regulatory effects on nucleosome assembly, chromatin structure, gene expression, DNA damage repair, cell cycle, mitochondrial function, and metabolic processes [2–4]. The disorder of Kglu modification is closely associated with many metabolic diseases, such as type 1 glutaric aciduria, diabetes, cancer, and neurodegenerative diseases [1, 5]. Therefore, accurate identification of Kglu sites is crucial for mastering the biological principles of proteins and exploring the molecular mechanisms of related diseases. Although traditional experimental methods have laid a good data foundation for the accumulation of Kglu data, their time-consuming and laborious shortcomings still cannot meet the needs of scientific development. Computational-based approaches for predicting PTM sites in proteins have drawn more attention as high throughput sequencing and machine learning(ML) have advanced [6].

Till now, over 10 computational-based approaches have been proposed to identify Kglu sites. For instance, amino acid factors(AAF), binary encoding (BE), and the composition of k-spaced amino acid pairs (CKSAAP) were both utilized to encode Kglu sites in Glut_Pred [7] and PUL-GLU [8], the difference between them is that they adopted different methods to solve the category imbalance problem. Position-Specific Propensity Matrix (PSPM) and Support Vector Machine(SVM) were used to support iGlu-Lys [9]. MDD_Glutar [10] considered the intrinsic dependence between substrate sites, grouped the data using maximal dependence decomposition (MDD), and constructed based on amino acid composition (AAC) and SVM. BiPepGlut [11] used sequential bi-peptide-based Position Specific Scoring Matrix (PSSM) feature for feature extraction, and Extra_tree for classification. RFGlutarySite [12] utilized 14 feature encoding methods with eXtreme Gradient Boosting (XGBoost) for feature selection and finally adopted Random Forest (RF) to construct the classifier. In addition to using different feature encoding techniques, iGlu_AdaBoost [13] and DEXGB_Glu [14] both take category imbalanced concerns into account. Some DL-based Kglu prediction models were proposed as deep learning (DL) advanced. For instance, iGluK-Deep [15] was proposed based on deep neural networks and Chou’s Pseudo Amino Acid Composition (PseAAC). ProtTrans-Glutar [16] incorporated the XGBoost and pre-trained features by Transformer. DeepDN_iGlu [17] was proposed by employing binary encoding as feature representation, using DenseNet as the classification model, and utilizing the focal loss function to address the imbalance issue. Deepro-Glu [18], as the latest Kglu prediction model, used the combination of pre-trained features obtained by ProtBert as well as four other manual features and introduced the attention mechanism in the MLP model. Details of these studies are summarized in Table 1.

**Table 1:** The cutting-edge ML-based Kglu prediction methods

Tool	features extraction/selection	balanced/classification algorithm	Performance parameters	AUC (%)	Acc (%)
GlutPred [7]	AAF + BE + CKSAAP mRMR + IFS	Bias SVM	ten-fold cross-validation	78.06%	74.90%
iGlu_Lys [9]	PSPM	SVM	ten-fold cross-validation	89.44%	88.38%
MDD_Glutar [10]	ACC	SVM	five-fold cross-validation	63.74%	61.60%
BiPepGlut [11]	bi-peptide-based PSSM	Extra-Trees	ten-fold cross-validation	—	74.58%
PUL-GLU [8]	AAF + BE + CKSAAP	Positive-unlabeled Learning/ SVM	ten-fold cross-validation	85.30%	81.50%
RFGlutarySite [12]	PseAAC + CT + SE + RE + IG + CTD + AAC + DC + TC + Autocorrelation、BE + AAindex + AAF + CKSAAP/Xgboost	Random Forest	ten-fold cross-validation	81.00%	72.30%
DEXGB_Glu [14]	AAindex, + ASA + SS + PSSM、RC、AC	Borderline-SMOTE/Xgboost	ten-fold cross-validation	—	87.09%
iGlu_AdaBoost [13]	188D + CKSAAP + EAAC	SMOTE-Tomek /Adaboost	ten-fold cross-validation	89.00%	79.98%
iGluK-Deep [15]	PseAAC	FCN	—	—	94.30%
ProtTrans-Glutar [16]	CTDD + EAAC + ProT5-XL-UniRef50	RUS/XGBoost	ten-fold cross-validation	70.75%	65.67%
DeepDN_iGlu [17]	BE	focal loss/DenseNet	ten-fold cross-validation	77.25%	66.00%
Deepro-Glu [18]	BE + DDE + BLOSUM62 + AAindex + ProtBert	Attention + MLP	ten-fold cross-validation	98.80%	96.30%

**Fig. 1:**
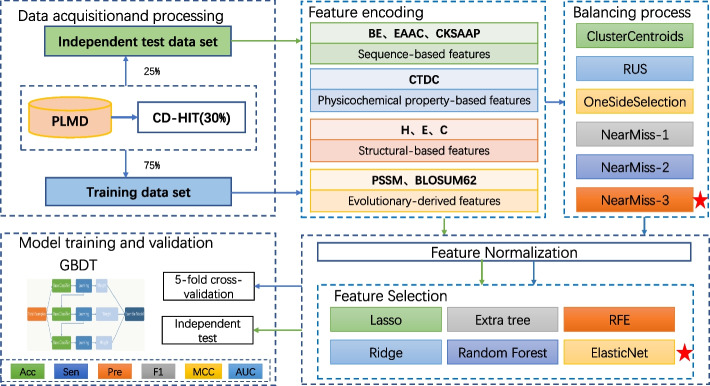
The GBDT_KgluSite schematic (The green arrows represent the independent test set's processing flow, and the blue arrows represent the training set's training flow. The ideal selection is denoted by the red pentagram.)

Although the above research on Kglu prediction have made active explorations in feature representation, feature selection, and model design, they still leave considerable room for improvement in terms of prediction performance. In this paper, we proposed a novel predictor named GBDT_KgluSite, which combined information on protein sequence, structure, physiochemistry, and evolution. Gradient Boosting Decision Tree (GBDT) was adopted for classification after NearMiss-3 and Elastic Net assisted in balancing the data and selecting the best features. The schematic diagram is shown in Fig. 1. The entire procedure may be divided into six steps, where the balancing strategy and training strategy are only used for training data.

## Results

### Sequence analysis

**Fig. 2:**
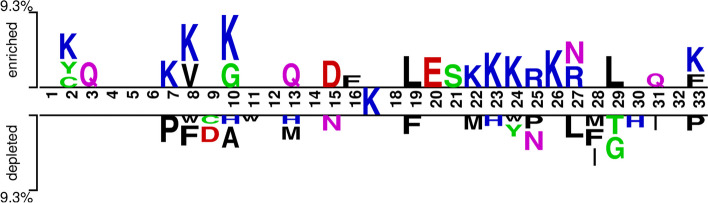
The Two-Sample Logo maps between positive and negative sequences

To determine whether the flanking sequence of Kglu sites might exhibit different patterns, the frequency of each amino acid around lysine in the positive and negative datasets was analyzed using a two-sample logo with t-test (*p* ≤ 0.05) [19]. Lysine (K) is highly enriched at several locations, including 2, 7, 8, 10, 22–24, 26, and 33 close to the Kglu sites, as shown in Fig. 2. Of fact, some amino acids are much more abundant than others, such as leucine (L), aspartic acid (D), and glutamic acid (E). On the other hand, certain locations downstream of the central Kglu sites are deficient in phenylalanine Phe (F), asparagine Asn (N), proline Pro (P), and methionine Met (M). This suggests that Kglu sites can be effectively distinguished using feature representation based on sequence information.

### Performance of GBDT_KgluSite model

To determine the optimal parameters of the model and avoid overfitting, grid search, and five-fold cross-validation were performed on the training dataset. Considering that the dataset is small and the feature dimensions are high, we only perform grid search for the three important parameters of GBDT, n_estimators, max_depth, and learning rate, and take the default values for the other parameters. Specifically, the training dataset was divided into five parts during each grid search, four of which were trained and one was tested in turn, and the average of the five results was finally used as the basis for comparison. The best model performance was finally obtained when the max_depth of GBDT was set to 6, the n_estimators was set to 200, and the learning rate was set to 1. Table 2 shows the results of five-fold cross-validation tests on the training dataset. As can be seen in Table 2 that the average values for Acc, Sen, Pre, F1, MCC, and AUC are 93.73%, 90.94%, 96.59%, 93.68%, 87.63%, and 98.14%, respectively. Additionally, their standard deviations are 1.14%, 1.39%, 0.97%, 1.16%, 2.25%, and 0.47%, which demonstrates the robustness of GBDT_KgluSite to some extent.

**Table 2:** Performance of GBDT_KgluSite on training dataset with five-fold cross-validation

Testing Set	Acc(%)	Sen(%)	Pre(%)	F1(%)	MCC	AUC(%)
1	94.92	92.27	97.66	94.89	89.98	98.71
2	92.09	88.95	95.27	92.00	84.39	97.42
3	93.22	90.61	95.91	93.18	86.59	98.11
4	93.79	90.61	97.04	93.71	87.78	98.11
5	94.63	92.27	97.09	94.62	89.39	98.33
Mean ± SD	93.73 ± 1.14	90.94 ± 1.39	96.59 ± 0.97	93.68 ± 1.16	87.63 ± 2.25	98.14 ± 0.47

Independent test data was utilized to verify the generalization of the GBDT_KgluSite, the results are shown in Table 3. Table 3 demonstrates GBDT_KgluSite's good generalization capabilities, with AUC values up to 96.75% and Sen values up to 95.06%.

**Table 3:** Performance of GBDT_KgluSite on independent test dataset

Testing data	Acc(%)	Sen(%)	Pre(%)	F1(%)	MCC	AUC(%)
1	90.11	95.06	85.08	89.79	80.73	96.75

### The effectiveness of different feature representation

**Fig. 3:**
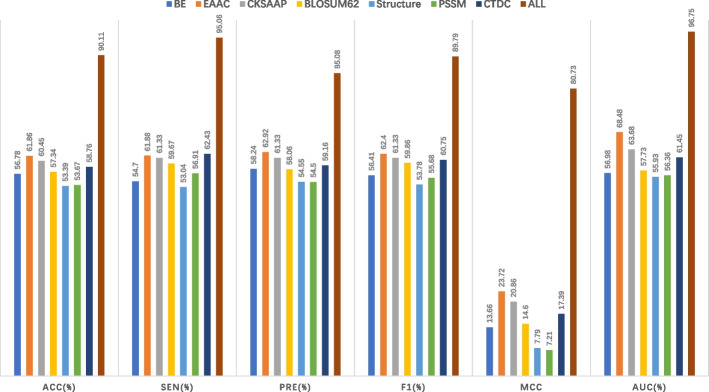
The performance comparison of different signal feature representation and feature combination

To verify the effectiveness of feature combination, we replaced the feature combination part of the GBDT_KgluSite model with each single feature respectively, kept the rest of the model unchanged, and obtained the prediction results of each single feature on the independent test data. Figure 3 displays the performance comparison among them.

As can be seen from Fig. 3, the AUC value represented by each feature is greater than 55%, indicating the effectiveness of the selected feature in predicting the Kglu position. Among them, EAAC features perform best, with an AUC value of 68.48%, while structural features have the lowest AUC value, with only 55.93% (Table S1). Structural characteristics are obtained through computational models rather than experimental validation, which may be the reason for its poor performance.

### The influence of feature selection techniques

**Fig. 4:**
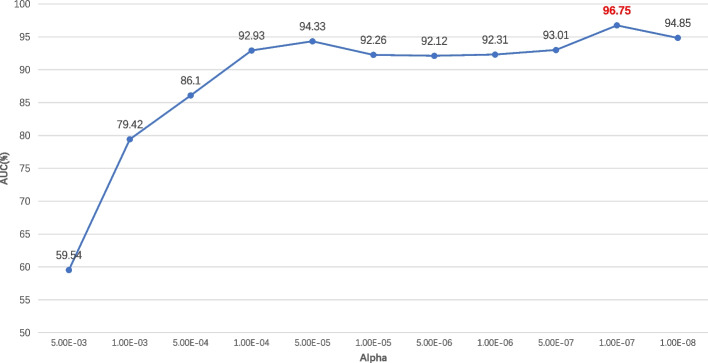
Performance of Elastic Net with different alpha values

**Fig. 5:**
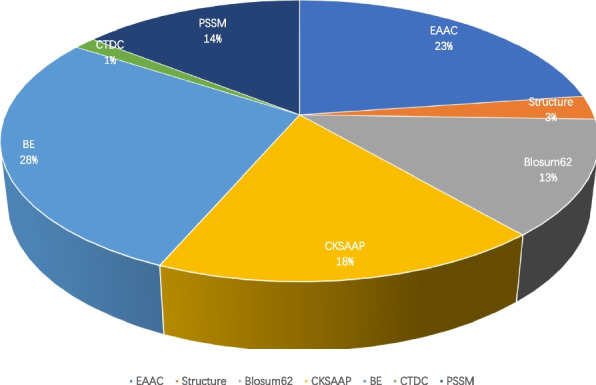
Proportional distribution of each feature after selection by Elastic Net

In this paper, the feature combination we utilized is 2895 dimension, which may result in overfitting as well as slowing down model training. Hence, it’s necessary to find a suitable feature selection method to solve this problem. In this paper, Elastic Net was used to select appropriate features, which combines the advantages of lasso and ridge regression by adding L1 and L2 penalty terms to the linear regression. The model performance following feature selection by Elastic Net with various alpha values is shown in Fig. 4 and Table S2. The model operates most effectively when the Alpha value is set to 0.000001, with the AUC value rising to 96.75%. The Elastic Net's feature selection yields a total of 2656 features, the makeup of which is depicted in Fig. 5. As can be observed from Fig. 5, EAAC, BE, and CKSAAP were accordingly the top three features.

**Fig. 6:**
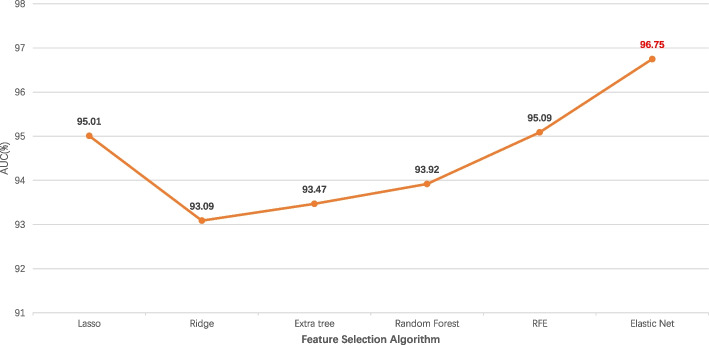
Comparison of AUC values with different feature selection algorithms

Additionally, we also compared the Elastic Net with several other top-notch feature selection techniques, including Lasso regression, Ridge regression, Extra tree, Random Forest(RF), and Recursive Feature Elimination (RFE) [20], the results are displayed in Fig. 6 and Table S3. Figure 6 demonstrates that Elastic Net achieves the highest AUC value, even though it only outperforms the second-ranked RFE by 1.69%. This not only represents Elastic Net's best performance in the aforementioned feature selection algorithm, but it also shows that the features employed in this paper can effectively convey different information.

### The influence of down-sampling techniques

Since the initial training dataset contains 530 positive samples and 3277 non-positive samples, which is imbalanced, there is a potential risk of directly training it to obtain the model, so it is necessary to construct a balanced training dataset with a suitable resampling technique. The resampling technique, which can be subdivided into over-sampling, down-sampling, and hybrid methods [21], is used to balance the proportion of positive and negative samples. Down-sampling is the frequently technique used in PTM field since positive samples in protein PTM data are experimentally proven and reliable datasets, however, negative samples may contain unidentified modification sites. To compare the effectiveness of different down-sampling methods, we kept the remaining modules of GBDT_KgluSite unchanged, and only changed the down-sampling technique module to obtain multiple models and compare the results of each model in the independent test data. Finally, the NearMiss-3 was chosen due to its superior performance when compared to the other five down-sampling methods, including Random under Sample (RUS), ClusterCentroids, OneSideSelection, NearMiss-1 and NearMiss-2 on the independent test data (Table 4). They were all implemented in the Python 3.7 imbalance-learn package (version 0.8.0).

**Table 4:** Performance of various down-sampling methods on independent test dataset

Model	Samples (N/P)	Acc (%)	Sen (%)	Pre (%)	F1 (%)	MCC	AUC (%)
ClusterCentroids	530/530	84.75	81.77	**87.57**	84.57	69.68	92.55
RUS	530/530	84.18	81.22	86.98	84.00	68.55	92.18
OneSideSelection	2863/530	82.20	80.66	83.91	82.25	64.47	90.21
NearMiss-1	530/530	83.62	80.11	86.83	83.33	67.48	93.11
NearMiss-2	530/530	85.31	83.43	87.28	85.31	70.71	92.00
NearMiss-3	530/530	**90.11**	**95.06**	85.08	**89.79**	**80.73**	**96.75**

To facilitate understanding, the highest value in each column is shown in bold. where the N and P in the Samples column brackets means negative and positive, respectively

As shown in Table 4, the performance of OnesideSelection is the lowest among these imbalance methods, probably because the data obtained by most of the methods can reach a balanced state, except for the ratio of data produced by the OnesideSelection method, which is still imbalanced (2863:530). While the NearMiss-3 method yields the highest overall model performance, outperforming the other methods in aomost most all metrics, with only slightly lower Pre values. Therefore, NearMiss-3 was selected as the imbalance strategy in this paper.

### Performance of different model classifiers

**Fig. 7:**
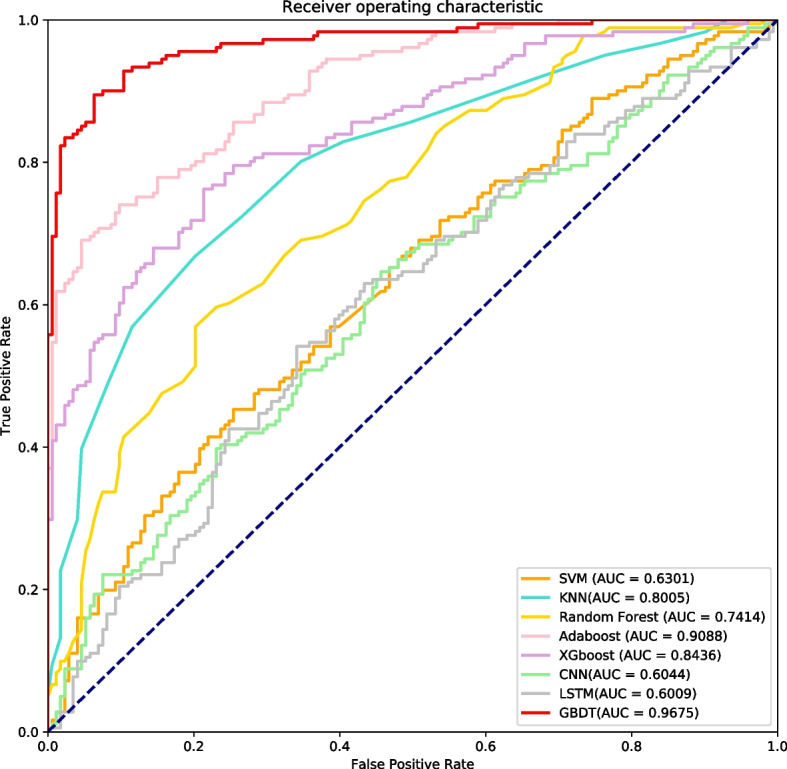
The ROC of different classifiers on the independent test dataset

To demonstrate the effectiveness of the GBDT algorithm proposed in GBDT_KgluSite, we kept the rest of the model unchanged and replaced the GBDT algorithm with SVM [22], RF [23], KNN(K-Nearest Neighbors), XGBoost [24], Adaboost(Adaptive Boosting) [25], CNN(Convolutional Neural Network) [26] and LSTM(Long Short Term Memory) [27], respectively. Figure 7 and Table S4 show the performance comparisons between the seven models and GBDT_KgluSite on the independent test data. As shown in Fig. 7, ensemble approaches generally outperformed other models in terms of overall performance. While deep learning models represented by LSTMs and CNNs perform poorly, this may be due to the fact that no suitable network structure has been built. The Acc, Sen, Pre, F1, MCC, and AUC for the GBDT_KgluSite outscored the second-ranked model Adaboost by 10.73%, 15.39%, 4.93%, 9.91%, 22.00, and 5.87%, respectively. As a whole, GBDT_ KgluSite shows better predictive performance across these ML models.

### Comparisons with state-of-the-art methods

**Fig. 8:**
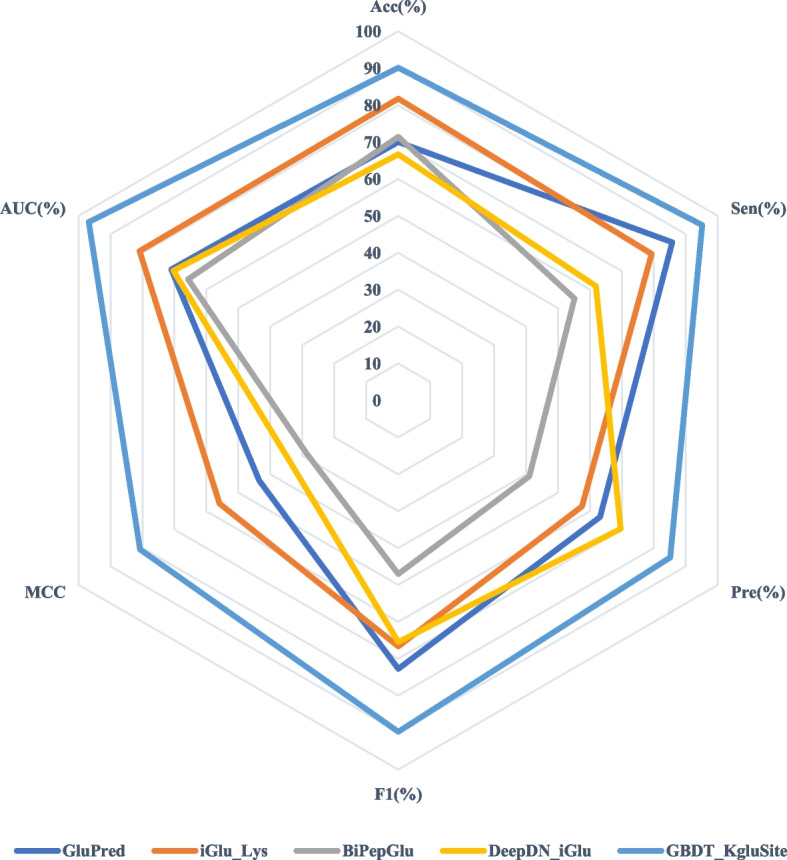
Radar plots of the performance comparison of different models in the independent test dataset

In the past few decades, several Kglu prediction models have been proposed, however, many of the relevant websites are inaccessible or the source code is difficult to reproduce. Hence, we compared GBDT_KgluSite with four available models on the independent test data (Table 5 and Fig. 8), namely GlutPred [7], iGlu_Lys [8], BiPepGlut [11], and DeepDN_iGlu [17].

**Table 5:** Performance of GBDT_KgluSite and other methods on the independent test dataset

Model	Acc (%)	Sen (%)	Pre (%)	F1 (%)	MCC	AUC (%)
GluPred [7]	70.00	85.71	63.16	72.73	43.44	70.98
iGlu_Lys [9]	81.74	79.31	57.50	66.67	55.87	80.89
BiPepGlu [11]	71.43	55.17	41.03	47.06	28.65	65.73
DeepDN_iGlu [17]	66.67	61.88	69.57	65.50	33.68	70.34
GBDT_KgluSite	**90.11**	**95.06**	**85.08**	**89.79**	**80.73**	**96.75**

To facilitate understanding, the highest value in each column is shown in bold

As shown in Table 5 and Fig. 8, GBDT_Kglusite far outperforms other methods in all metrics, especially MCC and AUC values, which were 24.86% and 15.86% higher than the second-ranked model, iGlu_Lys. This may due to insufficient features they used, such as Glut_Pred only incorporated three different types of amino acid sequence information (AAF, BE, and CKSAAP), while iGlu_ Lys only considered PSPM that represents sequence and special position information for amino acid pairs, BiPepGlu employed only PSSM, which indicated evolutionary information, and DeepDN_iGlu only considered the binary encoding.

## Discussion

We proposed GBDT_KgluSite, a novel Kglu site prediction model based on GBDT that achieved better prediction performance than that of previously established state-of-the-art models. Specifically, seven feature representation methods, including BE, BLOSUM62, EAAC, CKSAAP, CTDC, PSSM, and secondary structural information, were used to convert protein sequences into digital information firstly. The dataset-imbalance issue was addressed by the NearMiss-3, and redundant information in the features was filtered out by Elastic Net.

The experimental results show that GBDT_KgluSite has good robustness and generalization ability, with accuracy and AUC values of 93.73%, and 98.14% on five-fold cross-validation as well as 90.11%, and 96.75% on the independent test dataset, respectively. Meantime, we evaluated the efficacy of several feature selection algorithms, dataset-balancing techniques, and ML models through ablation experiments. The ablation experiment results indicate that the effective feature fusion of the above seven features and the application of GBDT make major contributions to the excellent performance of the GBDT_KgluSite, whereas feature selection methods and imbalanced handling strategy have auxiliary effects on improving model performance. Meanwhile, the ablation experiments further confirm the good results of Elastic Net and NearMiss-3 in terms of feature selection and imbalanced data processing effects.

Unfortunately, we also attempted to predict Kglu sites for other species by GBDT_ KgluSite, but the results were not ideal, thus this portion of the work was not presented in the paper. We speculate that this may be caused by the different distribution of amino acids of amino acid at Kglu sites in different species.

## Conclusions

In conclusion, we proposed the GBDT_KgluSite model for Kglu site prediction and demonstrated the combination of seven features with Elastic Net using GBDT has good stability and generality, which may help experimental scientists accelerate the discovery process of the Kglu sites in the protein. However, The GBDT_KgluSite model also has some shortcomings that need to be addressed, such as insufficient sample size, reliance on third-party libraries for feature representation (PSI-BLAST, PSRSM), failure to consider global features, and the unreliability of negative samples.

Therefore, we will conduct further research in the following directions to obtain more efficient and robust models. One direction would be to account for is to pay attention to the latest developments in Kglu site research and update the available dataset timely, as there are still few experimental validation data of Kglu compared to other PTM data, which restricts the performance of computational-based prediction methods to some extent. Also, the imbalance of datasets and the unreliability of negative datasets are two bottlenecks that constrain the development of this field. Although they can be ameliorated to some extent by existing imbalance strategies, they cannot solve the underlying problems. Therefore, we will try to introduce contrastive learning technique in the field of PTM, which does not rely on negative samples and has been applied with satisfactory results in the field of drug-target interactions [28]. Additionally, we will work on creating more appropriate and general feature representation methods, such as global feature representation that take protein–protein interaction information into account, as well as pre-training methods based on Natural Language Processing (NLP), such as Transformer or Large Language Model (LLM). Finally, developing an interactive website is our future work that needs to be completed.

As more Kglu site are experimentally validated and new feature representation method is proposed, data-driven computational methods, such as our model, will become even more effective and make significant contributions to the field of PTM research.

## Methods

### Data collection and pre-processing

The dataset was retrieved from the Protein Lysine Modification Database (PLMD) [29], which includes *Mus musculus* and *M. tuberculosis* Kglu proteins collected from two previous studies [1, 4]. We initially obtained 715 Kglu sites from 211 proteins from the PLMD database, and then used CD-hit to remove similar sequences with 30% sequence identity to obtain 208 non-redundant proteins, of which 707 Kglu sites were used as positive samples and 4,369 non-Kglu sites were used as non-positive samples.

The proteins were then transformed into Kglu-centered peptide sequences according to Chou's PseAAC [30], the details are as illustrated below:

$$\mathrm{P}={\mathrm{A}}_{-\mathrm{n}}\dots {{\mathrm{A}}_{-2}\mathrm{A}}_{-1}{\mathrm{KA}}_{1}{\mathrm{A}}_{2}\dots {\mathrm{A}}_{\upvarepsilon }$$

where K stands for the’Lysine’ amino acid and’A’ for the amino acid residues surrounding K, enotes the $\upvarepsilon$ th amino acid on the right side of K and ${\mathrm{A}}_{-\mathrm{n}}$ denotes the left one. Generally, n and $\upvarepsilon$ take the same value to obtain a peptide of length 2n + 1 with K as the center point. In this paper, each peptide segment's length was set at 33, and the placeholder residue "X" was employed to fill in the gaps.

Finally, the 707 Kglu peptides and 4369 non-Kglu peptides were divided into two groups: 25% were used as the independent test dataset and 75% as the training dataset (Table S5). However, the training dataset obtained here is imbalanced, consisting of 530 positive samples and 3277 non-positive samples. Therefore, it is necessary to use down-sampling techniques to balance the training dataset. The detail of the independent test dataset and the final training data set is displayed in Table 6. The dataset is available at https://github.com/flyinsky6/GBDT_KgluSite.

**Table 6:** Details of training data and independent test dataset

Datasets	All	Positive	Negative
Training dataset	1060	530	530
Independent test dataset	1269	177	1092

### Feature representation

The performance of the model depends on an effective feature representation. In this paper, seven feature representations were extracted for the benchmark data sets, namely Binary encoding (BE), Enhanced Amino Acid Composition (EAAC), the Compositon of K-Spaced Amino Acid Pairs (CKSAAP), the Composition of Composition,Transition, and Distribution (CTDC), Block Substitution Matrix 62 (BLOSUM62), structural feature, and Position-Specific Scoring Matrix (PSSM). These features belong to four categories respectively, and the details are as follows.

### Sequence based-features

#### Binary encoding

In this paper, we employed binary encoding (BE) to transform each peptide residue into one-hot code of length 22 (including 20 common amino acids and selenocysteine 'U', and pseudo-residue 'X'). Take the "ACDEFGHIKLMNPQRSTVWYUX" as an example, the letter A, C, and X are represented as 10000000000000000000, 010000000000000000, and 00000000000000000001, respectively. Thus, we can use a BE feature vector with a length of 726 to represent the 33-amino acid peptide fragment used in this paper.

#### EAAC

The EAAC encoding was created first by fixed-length sliding windows that move from the N- to C-terminus of each peptide, and then the frequency of every amino acid in the window was calculated using Equation:

$$f\left(t,win\right)=\frac{N(t,win)}{N(win)},t\in \left\{A,C,D,\dots ,Y\right\},win\in \{window1,window2,\dots ,window17\}$$

where N (t, win) is the amount of amino acid type t present in the sliding window win and N (win) is the size of the sliding window. The parameters are set to the default value of 5, resulting in a feature length of 581 for EAAC features, which are retrieved using iLearn [31].

#### CKSAAP

The CKSAAP coding scheme consists of k-spaced residue pairs (separated by k amino acids) in a peptide segment which has been used to predict PTM [32, 33], and extracellular matrix proteins [34]. For instance, there are 484 pairings of 22 amino acid combinations (i.e., AA, AC, …, XX). As a result, the CKSAAP descriptor can be defined as follows when K takes a particular value:

$$\{\frac{{N}_{AA}}{{N}_{total}},\frac{{N}_{AC}}{{N}_{total}},\dots ,\frac{{N}_{XX}}{{N}_{total}}\}$$

where N_total_ is the total amount of k-space residue pairs in the fragment, N_AA_ is the number of amino acid pairs AA in the fragment, and so on. In this paper, we chose K = 0 to obtain a 484-dimensional CKSAAP feature vector.

### Physicochemical property-based feature

#### CTDC

The amino acid distribution patterns of a certain structural or physicochemical attribute in a protein or peptide sequence are represented by Composition, Transition, and Distribution (CTD) features. Hence, CTDC means the composition feature. 13 types of physicochemical properties have been previously used for computing these features, such as hydrophobicity, and solvent accessibility [35]. Taking the hydrophobicity attribute as an example, all amino acids are divided into three groups: polar, neutral, and hydrophobic. The CTDC can be determined as follows:

$$C\left(r\right)=\frac{N(r)}{N}, r\in \{polar,neutral,hydrophobic\}$$

where $N$ is the sequence length, $N(r)$ is the amount of amino acid type $r$.

### Structure-based feature

The term "secondary structure" describes the particular conformation that results from the coiling or folding of the polypeptide backbone atoms along a particular axis, or the spatial arrangement of the backbone atoms of the peptide chain. It has one irregular secondary structure type, the coil region, along with two regular secondary structure states, the -helix (H), and -strand (E) (C). Structural information has been applied to several types of PTM prediction, including succinylation [36], ubiquitination [37], and malonylation [38]. However, as far as we are aware, it has not yet been used for Kglu's prediction. Although models such as Alphabold2 accelerate the growth of the field of protein structure, they are still unable to predict some overly long proteins. In this paper, we decided to employ PSRSM [39], which is rated as the best secondary structure predictor of protein [40] and can collect the secondary structure information of all proteins. The structural information's four letters, "C," "H," "O," and "X," can be represented by two binary numbers. Finally, each peptide fragment has a secondary structure of 66 dimensions.

### Evolutionary-derived information

#### PSSM

The PSSM is a matrix of L*20 (L stands for the length of protein amino acids, and 20 is the type of amino acids), and it was obtained by PSI-BLAST [41] after two iterations of sequence similarity search in the non-redundant (NR) database. The PSSM contains information on the conservativeness of amino acids because the element ${P}_{ij}$ in the matrix indicates the probability that the amino acid at position *i* of the sequence mutates into the *j*th amino acid during the evolutionary process. Positive values represent higher probabilities, whereas negative values represent lower probabilities.

The PSSM is a matrix of L*20 (L represents the length of protein amino acids, and 20 corresponds to the type of amino acids) obtained by two iterations of sequence similarity search in the non-redundant (NR) database by PSI-BLAST [41]. The element ${P}_{ij}$ in the matrix indicates the probability that the amino acid at position *i* of the sequence mutates into the *j*th amino acid during the evolutionary process, and if the value is positive, it indicates a higher probability, and vice versa, it indicates a lower probability, so the PSSM contains information on the conservativeness of amino acids. PSSM has been successfully applied in several fields of bioinformatics, including protein–protein interaction prediction [42], protein structure prediction [43], DNA–protein binding [44], and protein post-translational modification site prediction [14, 45, 46].

#### BLOSUM62

As the most used amino acid substitution matrix and the default matrix for comparing protein sequences in BLAST, BLOSUM62 (Blocks Substitution Matrix) is a scoring matrix for amino acid substitutions used in bioinformatics when comparing sequences [47]. They observed and measured the protein families in highly conservative sequences (the identity between sequences is greater than a predetermined threshold) from the BLOCKS database to sort out the probability of amino acid substitution, and then used the logarithm to determine the score in the matrix. Among them, the BLOSUM62 matrix is obtained with an identity greater than or equal to 62%.

### Feature normalization

The seven features included in this paper span a variety of value ranges. for instance, structural features all take the value 0 or 1, whereas the PSSM feature’s value ranges from -15 to 13. MinMaxScaler method from the scikit-learn processing package (version 1.0.2) was used to equalize the feature value ranges per column because the large disparity has a significant impact on the model's performance.

### Feature selection

High feature dimensions make the model more complex and raise the chance of overfitting. A crucial method for separating the useful features from the original characteristics and enhancing the efficiency of the learning algorithm is feature selection [20]. In this paper, Elastic Net was used for feature selection because it combines the advantages of Lasso and Ridge methods, with good stability and sparsity. The dataset's feature dimension was decreased from 2895 to 2656 after Elastic Net was used.

### Balancing method

The initial positive and negative sample ratio is greater than 1:6, which is indicative of an imbalanced data set, and may impacts model performance if used directly. Therefore, it is necessary to select an appropriate balanced data set method. The data set balance method in this paper is NearMiss-3. The primary concept of NearMiss is to establish a set of rules to separate the sample corresponding to the tiny class sample from the majority sample, which alleviates the problem of information loss to some extent. NearMiss is separated into NearMiss-1, NearMiss-2, and NearMiss-3 based on several rules. NearMiss-1 sampling rules for selecting the nearest K minority class samples’ average distance to the nearest majority class samples. NearMiss-2 sampling rules for selecting the majority class samples that are closest to the average distance of the farthest K minority class samples. To make sure that each niche sample is surrounded by mass samples, NearMiss-3 selects the K closest mass samples for each niche sample [48]. After numerous ablation experiments, it was discovered that NearMiss-3 performed better than other down-sampling strategies, hence it is utilized in this paper as down-sampling strategy.

### GBDT

GBDT, also referred to as MART (Multiple Additive Regression Tree), is an additive model based on the boosting strategy in which CART(Classification And Regression Tree) is used as the base classifier, and the forward distribution algorithm is adopted for greedy learning during training, and the CART tree is learned at each iteration using gradient descent to fit the residuals of the prior t-1 tree at each iteration to fit the residuals between the predicted results of the previous t-1 trees and the real values of the training samples, and finally accumulate the results of all trees as the final result [49]. In GBDT, numerous nonlinear transformations have powerful expressive capabilities and typically don't call for intricate feature engineering and feature transformation.

### Prediction assessment

**Fig. 9:**
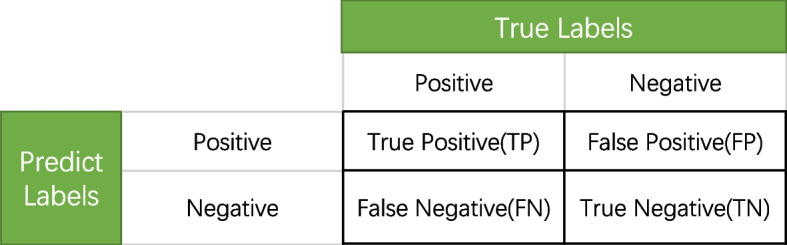
The confusion Matrix of binary Classification

Since predicting Kglu modification sites is a binary classification problem, we used the five traditional evaluation indicators of accuracy (Acc), sensitivity (Sen), precision (Pre), Matthew's correlation coefficient (MCC), and F1 score which are obtained from the confusion matrix (Fig. 9) [50] to assessment the performance of the model.

The details of the five evaluation indicators description are as follows:

1 $$\mathrm{Acc}=\frac{\mathrm{TP}+\mathrm{TN}}{\mathrm{TP}+\mathrm{FP}+\mathrm{TN}+\mathrm{FN}}$$

2 $$\mathrm{Sen}=\frac{\mathrm{TP}}{\mathrm{TP}+\mathrm{FN}}$$

3 $$\mathrm{Pre}=\frac{TP}{TP+FP}$$

4 $$\mathrm{F}1=\frac{2\times Pre\times Sen}{Pre+Sen}$$

5 $$\mathrm{MCC}=\frac{\left(\mathrm{TP}\times \mathrm{TN}\right)-(\mathrm{FP}\times \mathrm{FN})}{\sqrt{\left(\mathrm{TP}+\mathrm{FN}\right)\times \left(\mathrm{TN}+\mathrm{FP}\right)\times \left(\mathrm{TP}+\mathrm{FP}\right)\times (\mathrm{TN}+\mathrm{FN})}}$$

Accuracy is the proportion of all correctly predicted samples to the total sample;

Precision is the proportion of true positive samples among the samples that are predicted to be true; Recall is the proportion of positive samples that are predicted to be true; F1 score is the sum of precision and recall, which is closer to the smaller of the two quantities; MCC is essentially a correlation coefficient between the actual label and the predicted label [50].

The receiver operating characteristic (ROC) curve and the area under the ROC curve are also used to illustrate model performance. Among them, the ROC curve is shown on a graph with sensitivity as the vertical axis and 1-specificity as the horizontal axis, by various thresholds. The area below the ROC curve defined by the coordinate axis is known as the AUC. A more effective algorithm has a higher AUC value [51].

### Supplementary Information

**Additional file 1: Table S1.** Performance of different feature representation.

**Additional file 2: Table S2.** Performance of elastic net with different alpha values.

**Additional file 3: Table S3.** Comparison of AUC values with different feature selection algorithms.

**Additional file 4:** **Table S4.** The performance of different classifiers on the independent test data.

**Additional file 5:** **Table S5.** The details of dataset (negative).

## Data Availability

All data used in this paper can be freely downloaded https://github.com/flyinsky6/GBDT_KgluSite/Data, the data generated during analyzing are attached in the supplementary information files.

## Abbreviations

GBDT_KgluSite: Gradient-Boosting Decision Tree Lysine glutarylation Site
PTM: Post-Translational Modification
SVM: Support Vector Machine
BE: Binary Encoding
EAAC: Enhanced Amino Acid Composition
CKSAAP: Compositon of K-Spaced Amino Acid Pairs
DDE: Dipeptide Deviation from Expected mean
CTDC: Composition of Composition, Transition, and Distribution
BLOSUM62: Block Substitution Matrix 62
PSSM: Position-Specific Scoring Matrix
RF: Random Forest,
KNN: K-Nearest Neighbors
XGBoost: EXtreme Gradient Boosting
Adaboost: Adaptive Boosting
CNN: Convolutional Neural Network
RFE: Recursive Feature Elimination
RUS: Random Under Sample
ROC: Receiver Operating Characteristic
AUC: Area Under Curve
MCC: Matthew's Correlation Coefficient
NLP: Natural Language Processing
LLM: Large Language Model
